# Development and Validation of a Nomogram to Predict the Individual Future Stroke Risk for Adult Patients With Moyamoya Disease: A Multicenter Retrospective Cohort Study in China

**DOI:** 10.3389/fneur.2021.669025

**Published:** 2021-05-13

**Authors:** Fei Ye, Tianzhu Wang, Haoyuan Yin, Jiaoxing Li, Haiyan Li, Tongli Guo, Xiong Zhang, Tingting Yang, Liang Jie, Xiaoxin Wu, Qi Li, Wenli Sheng

**Affiliations:** ^1^Department of Neurology, The First Affiliated Hospital, Sun Yat-sen University, Guangzhou, China; ^2^Guangdong Provincial Key Laboratory of Diagnosis and Treatment of Major Neurological Diseases, The First Affiliated Hospital, Sun Yat-sen University, Guangzhou, China; ^3^Department of Neurology, The First Affiliated Hospital of Chongqing Medical University, Chongqing, China; ^4^Department of Neurosurgery, The First Affiliated Hospital of Jilin University, Changchun, China; ^5^Department of Neurology, The Third Affiliated Hospital, Sun Yat-sen University, Guangzhou, China; ^6^Department of Neurology, The Affiliated Hospital of Guizhou Medical University, Guiyang, China; ^7^Department of Neurology, The Second Affiliated Hospital of Chongqing Medical University, Chongqing, China; ^8^Department of Neurology, The Third Affiliated Hospital of Chongqing Medical University, Chongqing, China

**Keywords:** moyamoya disease, nomogram, risk factors, future stroke, translational medicine

## Abstract

**Background:** Studies exploring the predictive performance of major risk factors associated with future stroke events are insufficient, and a useful tool to predict individual risk is not available. Therefore, personalized advice for preventing future stroke in patients with moyamoya disease (MMD) cannot provide evidence-based recommendations. The aim of this study was to develop a novel nomogram with reliable validity to predict the individual risk of future stroke for adult MMD patients.

**Methods:** This study included 450 patients from seven medical centers between January 2013 and December 2018. Follow-ups were performed via clinical visits and/or telephone interviews from initial discharge to December 2019. The cohort was randomly assigned to a training set (2/3, *n* = 300) for nomogram development and a test set (1/3, *n* = 150) for external validation. The Kaplan-Meier analyses and receiver operating characteristic (ROC) curves were applied to assess the clinical benefits of this nomogram.

**Results:** Diabetes mellitus, a family history of MMD, a past history of stroke or transient ischemic attack, clinical manifestation, and treatment were identified as major risk factors via the least absolute shrinkage and selection operator (LASSO) method. A nomogram including these predictors was established via a multivariate Cox regression model, which displayed excellent discrimination [Harrell's concordance index (C-index), 0.85; 95% confidence interval (CI): 0.75–0.96] and calibration. In the external validation, the nomogram was found to have good discrimination (C-index, 0.81; 95% CI: 0.68–0.94) and calibration. In the subgroup analysis, this predictive nomogram also showed great performance in both ischemic-type (C-index, 0.90; 95% CI: 0.77–1.00) and hemorrhagic-type MMD (C-index, 0.72; 95% CI: 0.61–0.83). Furthermore, the nomogram was shown to have potential in clinical practice through Kaplan-Meier analyses and ROC curves.

**Conclusions:** We developed a novel nomogram incorporating several clinical characteristics with relatively good accuracy, which may have considerable potential for evaluating individual future stroke risk and providing useful management recommendations for adult patients with MMD in clinical practice.

## Introduction

Moyamoya disease (MMD) is a rare chronic cerebrovascular disease characterized by progressive stenosis and/or occlusion of the distal internal carotid artery (ICA) and its major branches with the development of abnormal collateral circulation at the base of the brain (1). Although genotype-phenotype correlation analysis has revealed that ring finger protein 213 (RNF213) is a key susceptibility gene for MMD, the etiology and pathogenesis are still not fully understood (2, 3). The incidence of MMD is high in Asian countries, and presenting symptoms are different across regions (4). MMD is an important cause of stroke and is associated with a relatively high recurrence risk of cerebrovascular events and a poor prognosis (5, 6). Several independent risk factors, including an initial symptom of intraventricular hemorrhage, smoking, Asian ethnicity, a history of transient ischemic attack (TIA), and a reduced hemodynamic reserve, have been reported to significantly contribute to recurrent stroke in MMD (7–9). However, the small sample sizes of these trials may cause inconsistencies across studies that engender heterogeneity. Therefore, a single risk factor might be insufficient to predict the individual probability of cerebrovascular events in the future.

Recent evidence has demonstrated that surgical revascularization is more effective in preventing recurrent strokes and improving neurological functions mainly for hemorrhagic MMD patients than conservative treatment (10–13). However, the recurrent stroke risk has not markedly decreased in ischemic MMD patients undergoing revascularization (14). In addition, whether surgical revascularization should be applied to asymptomatic patients and the optimal time to perform surgery in conservatively treated patients remain controversial (6). Thus, credible prognostic information must be obtained for individual patients to facilitate lifestyle and/or personalized therapeutic decisions. A nomogram is a visual scoring model based on several risk factors used to generate a numerical probability of a particular clinical event for an individual patient in terms of disease prognosis (15).

In this study, we first identify the major risk factors associated with future stroke events from all predictors. Second, we develop and validate a nomogram incorporating these risk factors to calculate individualized predictions of the future stroke risk and provide important treatment recommendations for adult patients with MMD.

## Materials and Methods

### Study Design

A multicenter retrospective cohort study was conducted to identify the major risk factors and develop a prediction model to evaluate the individual risk of future stroke in adult patients with MMD. The research was approved by all participating medical center's ethics committee (2020[138]). Patient consent was not required because of the use of deidentified data. This study was carried out on the basis of the Transparent Reporting of a multivariable prediction model for Individual Prognosis Or Diagnosis (TRIPOD) statement (16).

### Patient Selection

Patients with a clinical diagnosis of MMD were identified from the national health care system using the diagnosis-specific codes (ICD-9 code, 437.5; ICD-10 code, I67.5) in seven participating academic medical centers between January 2013 and December 2018. The inclusion criteria were as follows: (1) a diagnosis of MMD based on the 2012 Tokyo criteria (6) [definitive MMD was defined as the bilateral stenosis and/or occlusion of the terminal portion of ICAs and/or the proximal portion of the anterior cerebral artery (ACA) and/or the middle cerebral artery (MCA) with the abnormal vascular network via cerebral digital subtraction angiography (DSA) and/or magnetic resonance angiography (MRA); probable MMD was defined as the unilateral involvement with the abnormal vascular network near the lesion via DSA while lacking predisposing factors for steno-occlusive changes]; (2) age 18 years or older; (3) no history of prior use of antiplatelet agents; (4) no history of prior neurosurgery; and (5) complete data for all predictors of interest. Patients were excluded from the study if they were diagnosed with moyamoya syndrome, had malignant tumors or were lost to follow-up.

### Clinical Follow-Up

After initial discharge, long-term follow-ups were performed via clinical visits and/or telephone interviews until December 2019. The primary outcome was future stroke events defined as an acute focal infarction or hemorrhage of the brain, including sudden onset of a new focal neurological deficit and/or rapid worsening of an existing neurological deficit lasting 24 h or more with/without imaging evidence, while neurological dysfunctions lasting <24 h required imaging evidence by two independent neurologists. However, TIA, cognitive impairment, seizure, and perioperative stroke were excluded. The follow-up period was also obtained, which was defined as the time from initial discharge to the main outcome or December 2019.

### Predictors

The baseline characteristics of the MMD patients were collected from electronic medical records by two independent neurologists who did not participate in the study design and data analysis; the characteristics included mainly age at the first symptom, age at diagnosis, sex, ethnicity, vascular risk factors (hypertension, diabetes mellitus, hyperlipidemia, smoking, and drinking), a past history of stroke or TIA, a family history of MMD, modified Rankin Scale (mRS) score, bilateral steno-occlusive change, Suzuki stage, intracranial aneurysm, posterior cerebral artery (PCA) involvement, collateral circulation (17) [absent, ICA-vertebral artery (VA) originated, external carotid artery (ECA) originated], clinical manifestation [other, TIA, lacunar infarction, cerebral infarction, intracerebral hemorrhage (ICH), subarachnoid hemorrhage (SAH)], and treatment choice (conservative treatment, antiplatelet therapy, surgical revascularization). Hypertension was defined as the systolic blood pressure (BP) ≥ 140 mmHg and/or diastolic BP ≥ 90 mmHg, or prior to taking antihypertensive agents. Diabetes mellitus was defined as the fasting glucose ≥ 7.0 mmol/L and/or 2 h-oral glucose tolerance test (OGTT) ≥ 11.1 mmol/L, or prior to using the antidiabetic agents. Hyperlipidemia was defined as total cholesterol ≥ 5.17 mmol/L, triglyceride ≥ 2.3 mmol/L, and/or low-density lipoprotein cholesterol ≥ 1.8 mmol/L, or prior to using statins. Heavy smoking was defined as smoking ≥ 1 cigarette per day and more than 1 year. Alcohol consumption was defined as drinking alcohol > 200 g per week regularly. With regard to the treatment choice, surgical revascularization was recommended to patients with non-emergency status, markedly neurological symptoms and no surgical contraindications, including direct bypass, indirect bypass, and combined bypass. Antiplatelet therapy was recommended to patients with ischemic MMD and who were not willing to undergo revascularization, including aspirin (100 mg/d), clopidogrel (75 mg/d), or combined agents for the first 3 weeks followed by aspirin daily. Conservatively treated patients also included other medications and surgery but not revascularization. Additionally, if a patient presented with an initial symptom of TIA, we did not add to the past history of TIA. If data were missing, then the patient was excluded.

### Sample Size

An adequate sample size is required for nomograms to identify a significant effect estimate. If the primary outcome is binary, then the minimum frequencies of the two response levels must be more than 10 times the number of predictors according to Harrell's guidelines (18). In our study, a total of 19 predictors were considered. Thus, the sample size was at least 190 participants.

### Development of the Nomogram

The participants were randomly assigned to the training set and the test set by R Project: 2/3 of the patients were used to develop the individualized risk prediction model, while the remaining 1/3 of the patients were used to validate the nomogram. First, the least absolute shrinkage and selection operator (LASSO) method was used to identify major risk factors associated with future strokes from all the predictors (19). The major risk factors were filtered via non-zero coefficients (20). Then, a prediction model incorporating the selected factors was established by a multivariate Cox regression analysis (21). These selected factors were reported as hazard ratios (HRs) with 95% confidence intervals (CIs) and *P*-values. The Cox model is given by the equation:

Individual risk (future stroke event at Y years) = baseline hazard (time = Y) ^*^e^β0*X0+β1*X1+β2*X2+…^.

### Evaluation of the Nomogram

To evaluate the accuracy of the nomogram, discrimination and calibration were tested in this study. Discrimination was defined as the ability to distinguish patients with different outcomes. The Harrell's concordance index (C-index) was measured to evaluate this nomogram with bootstrapping validation (1,000 bootstrap resamples) (22). A higher value of the C-index indicated a more correct outcome of this nomogram, while a lower value suggested random chance. Then, the calibration was defined as the ability to reflect a difference between the predicted outcome and the actual outcome. Thus, a calibration plot was generated to present the predicted probabilities determined by the nomogram against the actual observed risk (23). An ideal prediction model would result in a 45-degree line showing perfect accuracy between predictive and observed outcomes. In addition, the absolute error of the nomogram was measured by the distance between an apparent plot and an ideal plot.

### Validation of the Nomogram

The independent test set was used for external validation (24). The C-index was calculated to estimate the performance of the nomogram in predicting outcomes with 1,000 bootstrap resamples. The calibration of the predictive nomogram was assessed based on a calibration plot in the external validation (25).

### Clinical Use of the Nomogram

Individual risk was calculated based on the prediction model in both the training set and the test set. Participants were classified into high-risk and low-risk groups according to the median value. The Kaplan-Meier analyses were applied to explore the stroke-free survival differences between both groups using the log-rank test (26). The receiver operating characteristic (ROC) curves were used to evaluate the 3-year performance of this nomogram (27).

### Statistical Analysis

SPSS statistical software version 26 (IBM Corp) and R Project for Statistical Computing version 4.0.2 were used for statistical analyses. Statistical significance was defined by a 2-sided *P*-value below 0.05. Continuous variables were calculated as the median with the standard deviation. Categorical variables were reported as the number with proportions. The *t*-test and Mann-Whitney *U* test were used to analyze differences in continuous variables. The Pearson chi-square test was performed to explore differences in categorical variables. The LASSO method was applied to identify major risk factors. Multivariable Cox regression analysis was used to develop the predictive nomogram. The analysis began in January 2020 and ended in September 2020.

## Results

### Clinical Characteristics and Main Outcomes

**Figure 1:**
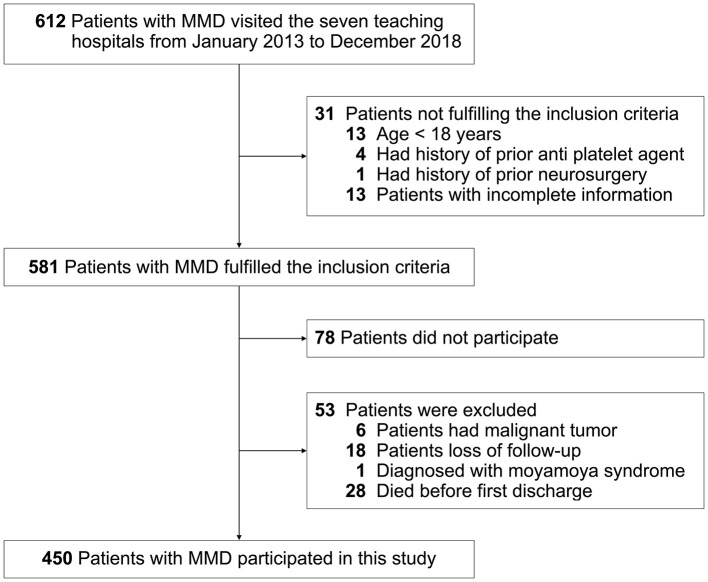
Flow chart presenting the process of patient inclusion and exclusion in this study.

This study was carried out as described in the flow chart (Figure 1). A total of 612 MMD patients were recruited at seven academic medical centers from January 2013 to December 2018. After applying the exclusion criteria, 450 patients (73.5%) who met the inclusion criteria and completed the clinical visits and/or telephone interviews through December 2019 were included in our final analysis (Supplementary Table 1). All patients were randomly assigned to either the training set (2/3 of the sample, *n* = 300; Supplementary Table 2) or the test set (1/3 of the sample, *n* = 150; Supplementary Table 3). Patient characteristics in both sets are provided in Table 1. No significant differences in clinical characteristics were found between the two datasets. Future stroke occurred in 46 patients (10.2%) during a follow-up of 34 ± 17 months in the present study. The detailed characteristics of these patients are summarized in Table 2. In addition, the demographic and clinical characteristics between patients with and without future stroke events are presented in Tables 3, 4. We found that a family history of MMD, a past history of stroke or TIA and treatment choice were remarkably different between both groups in the training and test sets, suggesting that these clinical features could be the potential risk factors for MMD with future strokes.

**Table 1:** The clinical characteristics of adult patients with moyamoya disease.

**Characteristic**	**All (*n =* 450)**	**Training (*n =* 300)**	**Test (*n =* 150)**	***P*-value**
Age (years)				
At first symptom	46 ± 11	46 ± 11	47 ± 12	0.229
At diagnosis	47 ± 11	47 ± 11	48 ± 12	0.265
Sex				
Female	231 (51.3%)	159 (53.0%)	72 (48.0%)	0.317
Male	219 (48.7%)	141 (47.0%)	78 (52.0%)	
Han ethnicity	443 (98.4%)	295 (98.3%)	148 (98.7%)	0.788
Vascular risk factors				
Hypertension	163 (36.2%)	107 (35.7%)	56 (37.3%)	0.729
Diabetes mellitus	56 (12.4%)	39 (13.0%)	17 (11.3%)	0.614
Hyperlipidemia	41 (9.1%)	28 (9.3%)	13 (8.7%)	0.817
Active smoking	133 (29.6%)	86 (28.7%)	47 (31.3%)	0.559
Alcohol consumption	93 (20.7%)	60 (20.0%)	33 (22.0%)	0.621
Past history of stroke or TIA	38 (8.4%)	27 (9.0%)	11 (7.3%)	0.549
Family history of MMD	25 (5.6%)	18 (6.0%)	7 (4.7%)	0.561
mRS score				
0	55 (12.2%)	35 (11.7%)	20 (13.3%)	0.916
1	264 (58.7%)	177 (59.0%)	87 (58.0%)	
2	69 (15.3%)	45 (15.0%)	24 (16.0%)	
3	20 (4.4%)	13 (4.3%)	7 (4.7%)	
4	22 (4.9%)	17 (5.7%)	5 (3.3%)	
5	20 (4.4%)	13 (4.3%)	7 (4.7%)	
Angiography findings				
Intracranial aneurysm	50 (11.1%)	30 (10.0%)	20 (13.3%)	0.289
Bilateral	408 (90.7%)	271 (90.3%)	137 (91.3%)	0.731
Suzuki stage				
I	14 (3.1%)	10 (3.3%)	4 (2.7%)	0.328
II	17 (3.8%)	13 (4.3%)	4 (2.7%)	
III	350 (77.8%)	226 (75.3%)	124 (82.7%)	
IV	51 (11.3%)	35 (11.7%)	16 (10.7%)	
V	16 (3.6%)	14 (4.7%)	2 (1.3%)	
VI	2 (0.4%)	2 (0.7%)	0 (0.0%)	
PCA involvement	69 (15.3%)	51 (17.0%)	18 (12.0%)	0.165
Collateral circulation				
ICA-VA originated	367 (81.6%)	239 (79.7%)	128 (85.3%)	0.340
ECA originated	67 (14.9%)	49 (16.3%)	18 (12.0%)	
Absent	16 (3.6%)	12 (4.0%)	4 (2.7%)	
Disease type				
Other	47 (10.4%)	31 (10.3%)	16 (10.7%)	0.962
TIA	63 (14.0%)	42 (14.0%)	21 (14.0%)	
Lacunar infarction	12 (2.7%)	7 (2.3%)	5 (3.3%)	
Cerebral infarction	112 (24.9%)	72 (24.0%)	40 (26.7%)	
ICH	140 (31.1%)	96 (32.0%)	44 (29.3%)	
SAH	76 (16.9%)	52 (17.3%)	24 (16.0%)	
Treatment				
Conservative treatment	266 (59.1%)	186 (62.0%)	80 (53.3%)	0.154
Antiplatelet therapy	62 (13.8%)	36 (12.0%)	26 (17.3%)	
Surgical revascularization	122 (27.1%)	78 (26.0%)	44 (29.3%)	
Follow-up (months)	34 ± 17	34 ± 17	35 ± 18	0.653
Future stroke events	46 (10.2%)	29 (9.7%)	17 (11.3%)	0.582

**Table 2:** Summary of 46 patients with recurrent stroke.

**No**	**Age at first symptom (years)**	**Sex**	**Vascular risk factor**	**Family history of MMD**	**Past history of stroke or TIA**	**mRS score**	**Suzuki stage**	**Clinical manifestation**	**Treatment**
**Training group (*****n=*** **29)**									
1	57	M	Hypertension, diabetes, smoking	None	Had	5	III	Cerebral infarction	Antiplatelet
2	41	F	Hypertension, hyperlipidemia	Had	Had	2	III	Cerebral infarction	Antiplatelet
3	60	F	Hypertension, diabetes	None	None	3	III	Cerebral infarction	Conservative
4	32	M	Hyperlipidemia	Had	None	1	IV	Cerebral infarction	Antiplatelet
5	39	M	None	None	None	3	III	Cerebral infarction	Conservative
6	22	F	None	None	None	2	III	Cerebral infarction	Conservative
7	24	F	None	None	None	1	III	ICH	Conservative
8	46	F	Hypertension	None	Had	1	III	ICH	Conservative
9	35	F	Hypertension, diabetes	None	None	0	III	SAH	Conservative
10	45	F	Diabetes	Had	None	4	III	ICH	Conservative
11	56	M	Hypertension, smoking, drinking	Had	None	1	V	Cerebral infarction	Antiplatelet
12	26	F	Hyperlipidemia	None	Had	0	II	Cerebral infarction	Conservative
13	25	M	Hypertension	None	Had	0	III	Cerebral infarction	Conservative
14	40	M	None	Had	Had	1	III	Cerebral infarction	Antiplatelet
15	40	F	None	None	Had	2	IV	ICH	Conservative
16	48	F	None	None	None	3	III	ICH	Conservative
17	57	F	Hypertension, diabetes	None	None	1	III	Cerebral infarction	Conservative
18	46	F	Hypertension	Had	Had	3	II	Cerebral infarction	Conservative
19	45	F	Hypertension, smoking	None	None	1	III	SAH	Conservative
20	36	M	None	Had	None	1	III	ICH	Conservative
21	49	M	Smoking	None	None	1	III	SAH	Conservative
22	53	M	Drinking	None	Had	1	III	ICH	Conservative
23	60	M	Smoking, drinking	None	Had	1	III	SAH	Surgical
24	20	M	None	Had	None	1	III	ICH	Conservative
25	36	M	None	None	None	2	III	ICH	Conservative
26	52	F	None	Had	None	1	III	ICH	Conservative
27	43	F	None	None	Had	3	IV	ICH	Conservative
28	58	M	Hypertension	None	Had	0	III	ICH	Conservative
29	36	M	None	None	None	4	V	ICH	Conservative
**Test group (*****n=*** **17)**									
1	38	M	Hyperlipidemia	None	None	2	I	ICH	Conservative
2	45	M	Hypertension, hyperlipidemia, smoking, drinking	Had	None	2	III	ICH	Conservative
3	54	F	Hypertension, diabetes	None	None	1	III	SAH	Conservative
4	33	M	Smoking, drinking	None	None	0	III	Cerebral infarction	Conservative
5	38	F	None	Had	None	2	III	Cerebral infarction	Conservative
6	48	F	None	None	Had	0	III	Cerebral infarction	Conservative
7	45	M	None	None	None	2	III	ICH	Conservative
8	36	F	None	None	None	0	III	SAH	Conservative
9	37	F	Diabetes	Had	Had	0	III	TIA	Surgical
10	35	F	None	Had	None	0	III	Other	Conservative
11	47	F	Hypertension, diabetes	None	None	3	III	ICH	Conservative
12	25	F	None	Had	Had	1	III	Cerebral infarction	Surgical
13	55	M	None	None	None	1	III	Cerebral infarction	Conservative
14	43	F	None	None	Had	5	III	SAH	Conservative
15	53	M	None	None	None	1	III	SAH	Surgical
16	52	M	Smoking, drinking	None	None	1	III	ICH	Conservative
17	26	M	None	Had	None	1	II	ICH	Conservative

**Table 3:** Comparison of demographic and clinical characteristics between patients with and without future stroke events in the training group.

**Characteristic**	**With (*n =* 29)**	**Without (*n =* 271)**	***P*-value**
Age (years)			
At first symptom	42 ± 12	46 ± 11	0.061
At diagnosis	44 ± 11	47 ± 11	0.231
Sex			
Female	15 (51.7%)	144 (53.1%)	0.885
Male	14 (48.3%)	127 (46.9%)	
Han ethnicity	29 (100.0%)	266 (98.2%)	0.461
Vascular risk factors			
Hypertension	11 (37.9%)	96 (35.4%)	0.789
Diabetes mellitus	5 (17.2%)	34 (12.5%)	0.475
Hyperlipidemia	3 (10.3%)	25 (9.2%)	0.844
Active smoking	5 (17.2%)	81 (29.9%)	0.152
Alcohol consumption	3 (10.3%)	57 (21.0%)	0.171
Past history of stroke or TIA	12 (41.4%)	15 (5.5%)	< 0.001
Family history of MMD	9 (31.0%)	9 (3.3%)	< 0.001
mRS score			
0	4 (13.8%)	31 (11.4%)	0.018
1	14 (48.3%)	163 (60.1%)	
2	3 (10.3%)	42 (15.5%)	
3	5 (17.2%)	8 (3.0%)	
4	2 (6.9%)	15 (5.5%)	
5	1 (3.4%)	12 (4.4%)	
Angiography findings			
Intracranial aneurysm	4 (13.8%)	26 (9.6%)	0.474
Bilateral	27 (93.1%)	244 (90.0%)	0.595
Suzuki stage			
I	0 (0.0%)	10 (3.7%)	0.826
II	2 (6.9%)	11 (4.1%)	
III	22 (75.9%)	204 (75.3%)	
IV	3 (10.3%)	32 (11.8%)	
V	2 (6.9%)	12 (4.4%)	
VI	0 (0.0%)	2 (0.7%)	
PCA involvement	5 (17.2%)	46 (17.0%)	0.971
Collateral circulation			
ICA-VA originated	24 (82.8%)	215 (79.3%)	0.512
ECA originated	5 (17.2%)	44 (16.2%)	
Absent	0 (0.0%)	12 (4.4%)	
Disease type			
Other	0 (0.0%)	31 (11.4%)	0.013
TIA	0 (0.0%)	42 (15.5%)	
Lacunar infarction	0 (0.0%)	7 (2.6%)	
Cerebral infarction	12 (41.4%)	60 (22.1%)	
ICH	13 (44.8%)	83 (30.6%)	
SAH	4 (13.8%)	48 (17.7%)	
Treatment			
Conservative treatment	22 (75.9%)	164 (60.5%)	0.044
Antiplatelet therapy	5 (17.2%)	31 (11.4%)	
Surgical revascularization	2 (6.9%)	76 (28.0%)	
Follow-up (months)	43 ± 20	33 ± 16	0.024

**Table 4:** Comparison of demographic and clinical characteristics between patients with and without future stroke events in the test group.

**Characteristic**	**With (*n =* 17)**	**Without (*n =* 133)**	***P*-value**
Age (years)			
At first symptom	42 ± 9	48 ± 12	0.036
At diagnosis	43 ± 9	49 ±12	0.040
Sex			
Female	9 (52.9%)	63 (47.4%)	0.665
Male	8 (47.1%)	70 (52.6%)	
Han ethnicity	17 (100.0%)	131 (98.5%)	0.611
Vascular risk factors			
Hypertension	3 (17.6%)	53 (39.8%)	0.075
Diabetes mellitus	3 (17.6%)	14 (10.5%)	0.383
Hyperlipidemia	2 (11.8%)	11 (8.3%)	0.630
Active smoking	3 (17.6%)	44 (33.1%)	0.196
Alcohol consumption	3 (17.6%)	30 (22.6%)	0.645
Past history of stroke or TIA	4 (23.5%)	7 (5.3%)	0.007
Family history of MMD	6 (35.3%)	1 (0.8%)	< 0.001
mRS score			
0	5 (29.4%)	15 (11.3%)	0.231
1	6 (35.3%)	81 (60.9%)	
2	4 (23.5%)	20 (15.0%)	
3	1 (5.9%)	6 (4.5%)	
4	0 (0.0%)	5 (3.8%)	
5	1 (5.9%)	6 (4.5%)	
Angiography findings			
Intracranial aneurysm	2 (11.8%)	18 (13.5%)	0.840
Bilateral	17 (100.0%)	120 (90.2%)	0.177
Suzuki stage			
I	1 (5.9%)	3 (2.3%)	0.425
II	1 (5.9%)	3 (2.3%)	
III	15 (88.2%)	109 (82.0%)	
IV	0 (0.0%)	16 (12.0%)	
V	0 (0.0%)	2 (1.5%)	
VI	0 (0.0%)	0 (0.0%)	
PCA involvement	0 (0.0%)	18 (13.5%)	0.106
Collateral circulation			
ICA-VA originated	16 (94.1%)	112 (84.2%)	0.200
ECA originated	0 (0.0%)	18 (13.5%)	
Absent	1 (5.9%)	3 (2.3%)	
Disease type			
Other	1 (5.9%)	15 (11.3%)	0.713
TIA	1 (5.9%)	20 (15.0%)	
Lacunar infarction	0 (0.0%)	5 (3.8%)	
Cerebral infarction	5 (29.4%)	35 (26.3%)	
ICH	6 (35.3%)	38 (28.6%)	
SAH	4 (23.5%)	20 (15.0%)	
Treatment			
Conservative treatment	14 (82.4%)	66 (49.6%)	0.027
Antiplatelet therapy	0 (0.0%)	26 (19.5%)	
Surgical revascularization	3 (17.6%)	41 (30.8%)	
Follow-up (months)	44 ± 25	34 ± 17	0.106

### The Major Risk Factors Associated With Future Stroke Events

**Figure 2:**
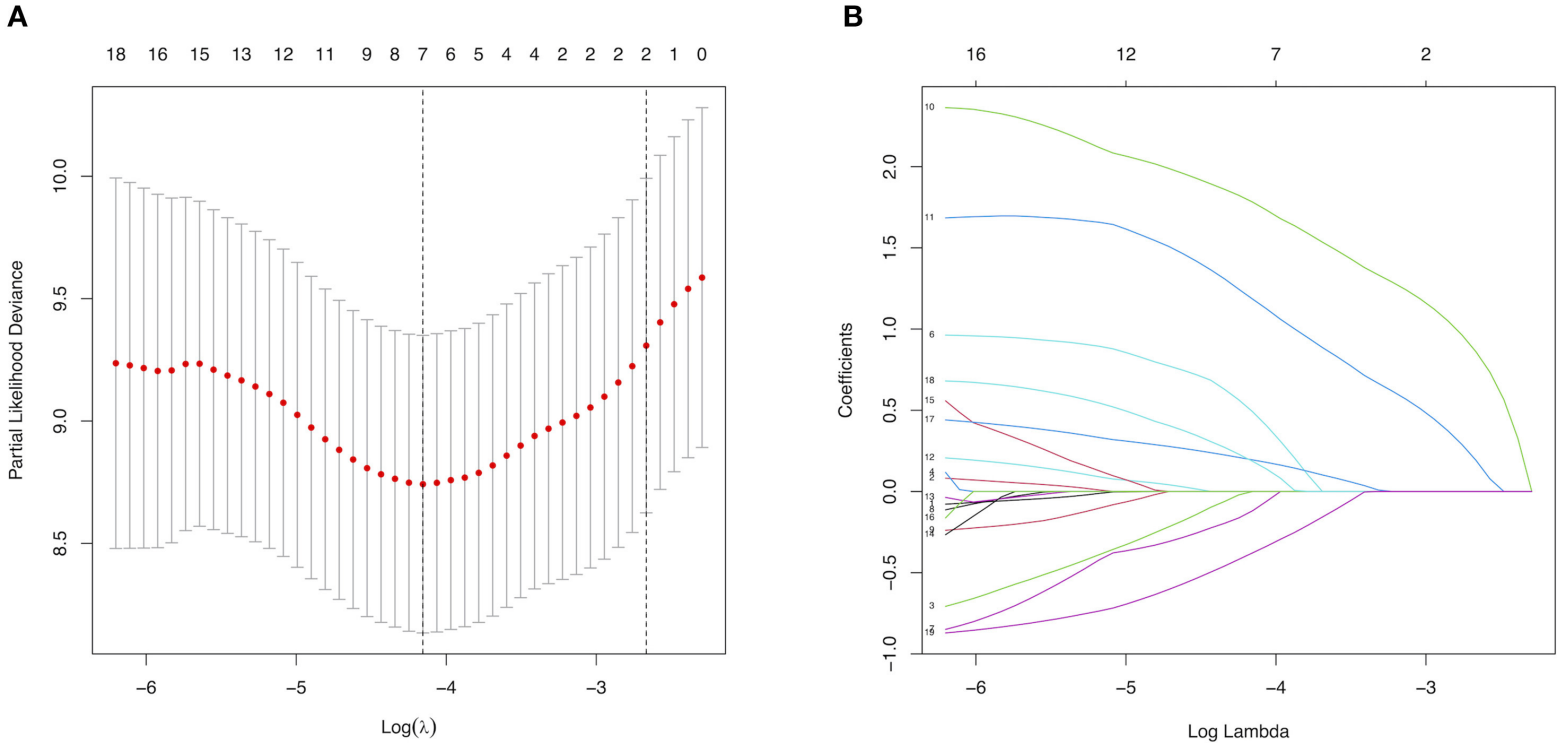
Selection of major risk factors using the least absolute shrinkage and selection operator (LASSO) binary logistic regression model. **(A)** Tuning parameter (λ) selection in the LASSO model used 5-fold cross-validation via minimum criteria. The partial likelihood deviance (binomial deviance) curve was plotted vs. log(λ). Dotted vertical lines were drawn at the optimal values using the minimum criteria and the 1 standard error of the minimum criteria (the 1-SE criteria). **(B)** LASSO coefficient profiles of the 19 predictors. A coefficient profile plot was produced against the log(λ) sequence. A vertical line was drawn at the value selected using 5-fold cross-validation, where the optimal λ resulted in 6 non-zero coefficients.

Among texture features, a total of 5 major risk factors with non-zero coefficients were selected from the 19 potential predictors in the 300 adult patients with MMD in the training set using the LASSO regression model (1:4 ratio, Figure 2), including diabetes mellitus, a family history of MMD, a past history of stroke or TIA, clinical manifestation, and treatment choice.

### Development of an Individualized Prediction Model

**Figure 3:**
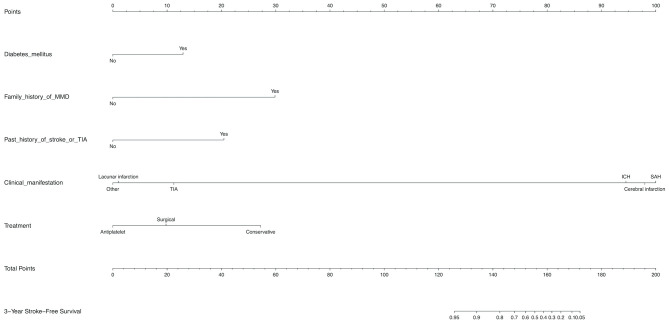
Development of a novel nomogram for predicting the individual risk of future stroke among adult patients with moyamoya disease (MMD) via a multivariate Cox regression analysis. The predictive nomogram was developed in the training set, with diabetes mellitus, a family history of MMD, a past history of stroke or TIA, clinical manifestation, and treatment incorporated.

Diabetes mellitus, a family history of MMD, a past history of stroke or TIA, clinical manifestation and treatment were all identified as independent risk factors by multivariable Cox regression analysis (Table 5). Therefore, a predictive nomogram incorporating the above predictors was developed for calculating the individual probability of 3-year stroke-free survival and is presented in Figure 3.

**Table 5:** The significant predictors of future stroke events in adult patients with moyamoya disease via a multivariate Cox regression analysis.

**Variable**	**HR**	**95% CI**	***P*-value**
Diabetes mellitus	3.54	1.23–10.20	0.019
Family history of MMD	10.51	4.23–26.12	< 0.001
Past history of stroke or TIA	5.98	2.43–14.70	< 0.001
Clinical manifestation	1.52	1.10–2.11	0.012
Treatment	0.38	0.17–0.84	0.017

### Performance of the Nomogram in Predicting the Individual Future Stroke Risk

**Figure 4:**
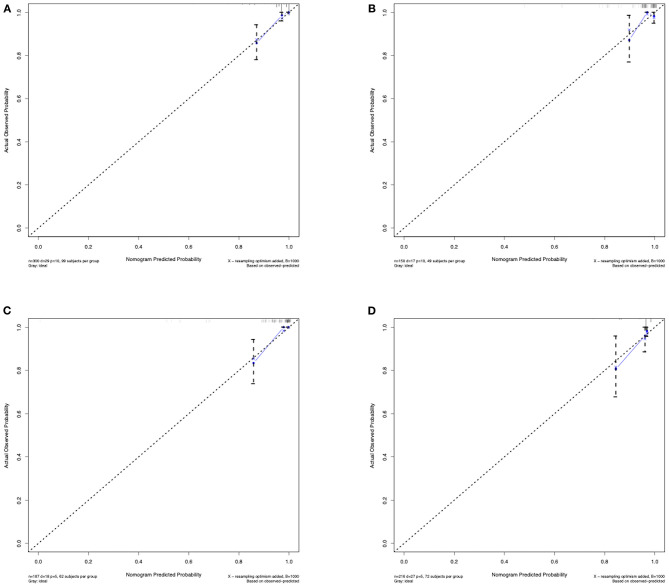
Calibration curves of the predictive nomogram. **(A)** Calibration curve of the predictive nomogram for 3-year stroke-free survival in the training set. **(B)** Calibration curve of the predictive nomogram for 3-year stroke-free survival in the test set. **(C)** Calibration curve of the predictive nomogram for 3-year stroke-free survival in the ischemic-type group. **(D)** Calibration curve of the predictive nomogram for 3-year stroke-free survival in the hemorrhagic-type group. Calibration curves depict the calibration of each model in terms of the agreement between the predicted risks of future stroke and observed outcomes of future stroke. The x-axis represents the predicted future stroke risk. The y-axis represents the actual observed rate of future stroke. The diagonal dotted line represents a perfect prediction by an ideal model. The solid line represents the performance of the nomogram, where a closer fit to the diagonal dotted line represents a better prediction.

The C-index for the predictive nomogram was 0.85 (95% CI: 0.75–0.96) by bootstrapping validation in the training set, indicating good discrimination of this nomogram. The calibration plot of this prediction model revealed good accordance between actual observations and nomogram predictions in the training set (Figure 4A). In this nomogram, the apparent performance exhibited high predictive power.

### Validation of the Nomogram in Predicting the Individual Future Stroke Risk

The C-index of the nomogram for predicting the individual future stroke risk was 0.81 (95% CI: 0.68–0.94) in the test set for external validation. Good calibration was also observed in the test set (Figure 4B).

### Clinical Use of the Nomogram

**Figure 5:**
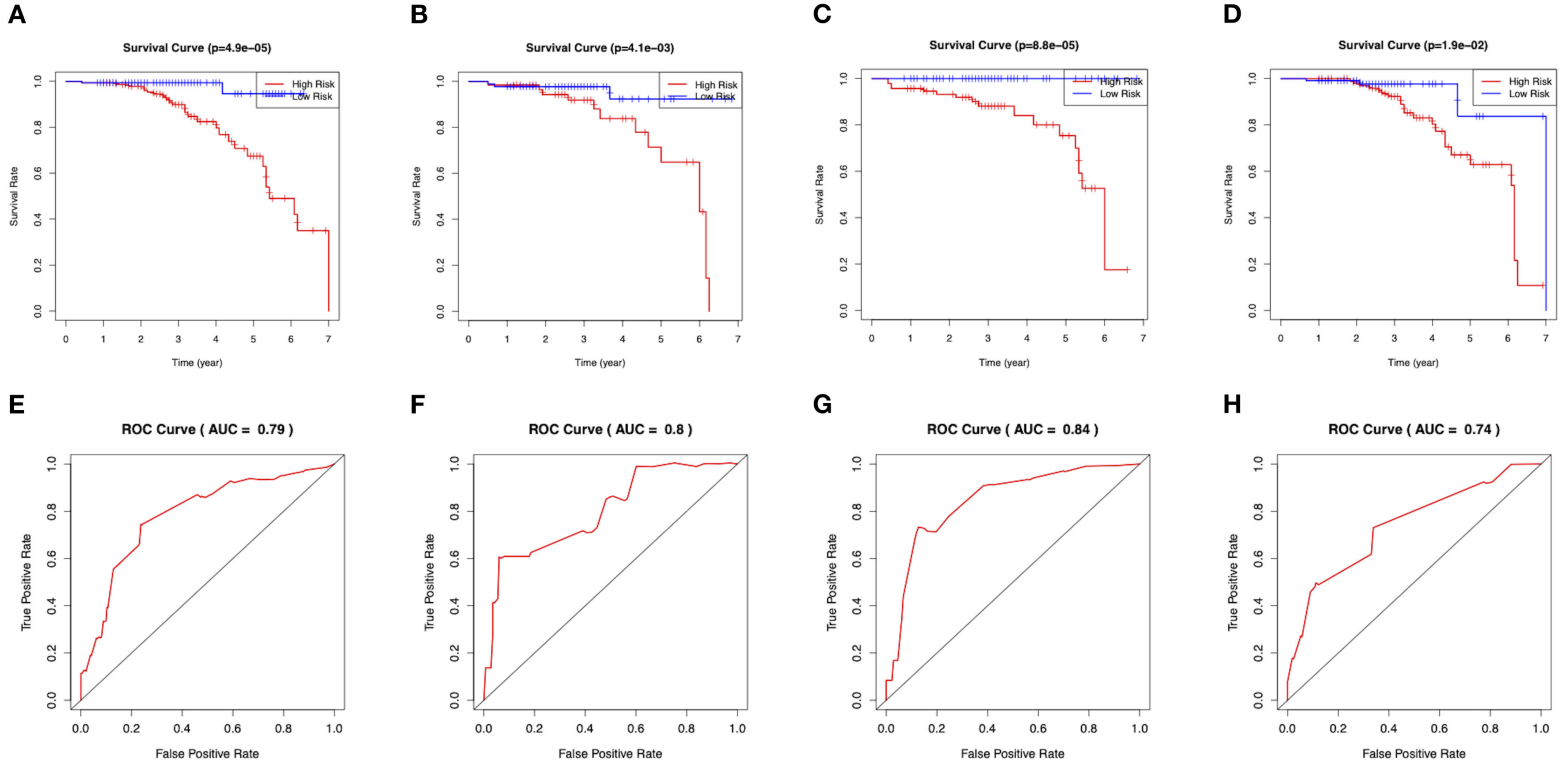
Evaluation of the clinical performance of the nomogram. **(A)** The Kaplan-Meier plot of the predictive nomogram in the training set. **(B)** The Kaplan-Meier plot of the predictive nomogram in the test set. **(C)** The Kaplan-Meier plot of the predictive nomogram in the ischemic-type group. **(D)** The Kaplan-Meier plot of the predictive nomogram in the hemorrhagic-type MMD. **(E)** ROC curves for predictions of 3-year stroke-free survival in the training set. **(F)** ROC curves for predictions of 3-year stroke-free survival in the test set. **(G)** ROC curves for predictions of 3-year stroke-free survival in the ischemic-type group. **(H)** ROC curves for predictions of 3-year stroke-free survival in the hemorrhagic-type MMD.

On the basis of the prediction model, patients were classified into high-risk and low-risk groups via a cutoff of mean value. Patients in the high-risk group suffered more future stroke risks than those in the low-risk group (Training: 18.0 vs. 1.3%; Test: 22.2 vs. 3.4%). The Kaplan-Meier plots also demonstrated that patients in the high-risk group were associated with poor stroke-free survival in Figures 5A,B. The 3-year area under the curve (AUC) values were 0.79 in the training set (Figure 5E) and 0.80 in the test set (Figure 5F). In general, this nomogram indicated a good predictive accuracy of the individual future stroke risk in clinical practice.

### Subgroup Analysis

In the subgroup analysis, we independently evaluated the performance of this nomogram in the 187 ischemic-type and 216 hemorrhagic-type MMD patients. Future stroke occurred in 18 ischemic-type patients (9.6%) during a follow-up of 33 ± 18 months and in 27 hemorrhagic-type patients (12.5%) during a follow-up of 35 ± 16 months (Supplementary Tables 4, 5). This predictive nomogram showed great performance in both ischemic-type (C-index, 0.90; 95% CI: 0.77–1.00) and hemorrhagic-type MMD (C-index, 0.72; 95% CI: 0.61–0.83) with excellent calibration (Figures 4C,D). Additionally, the individual risk score was calculated according to this nomogram, which was used to divide these patients into high-risk and low-risk groups on the basis of the mean value. The survival analyses showed that the stroke-free survival was significantly higher in the low-risk group in both ischemic-type and hemorrhagic-type groups (Figures 5C,D). The ROC curves also demonstrated good 3-year AUC in the ischemic-type group (AUC = 0.84) and hemorrhagic-type group (AUC = 0.74) (Figures 5G,H). In short, this nomogram could be independently used in different MMD subgroups with a great predictive ability.

## Discussion

In this study, the prediction model incorporating major risk factors performed well in predicting individual future stroke risk in adult MMD patients with relatively good accuracy. Additionally, the model may act as an available scoring system to distinguish the risk level and help doctors to provide treatment recommendations. Although many observational studies have revealed the clinical features and therapeutic methods for MMD, few have focused on the risk factors involved in prognosis. These findings from previous studies were not unique due to small sample sizes. This study was designed with a total of 450 patients from seven centers to acquire reliable results.

Similar to previous studies, our study has demonstrated that a pre-existing history of stroke or TIA might be an independent risk factor associated with future stroke events. Except for chronic steno-occlusive changes of the circle of Willis, Lee et al. (28) has revealed autoregulatory dysfunctions of BP in MMD patients, which could cause a fast or persistent cerebral ischemia symptom. However, the proportion of patients with a pre-existing history of stroke or TIA in our study was distinctly lower than the proportion in the study by Hervé et al. (8). Furthermore, patients with TIA could not be recorded because such mild symptoms that manifested with a rapidly recovery were easily ignored by patients; therefore, it is likely that this could explain the difference. In addition, the clinical manifestation was identified as a risk factor in this study. Hemorrhagic symptoms were the most common disease type in adult MMD patients with a slight advantage over ischemic symptoms. The hemorrhagic type of ICH and SAH constitute an important factor associated with a relatively higher risk of future strokes in both our study and the study by Kim et al. study (7). ICH originated from the abnormal moyamoya vessels due to the developmental functional disorders of vascular endothelial cells (29). Hemorrhage sites from the posterior circulation have a higher recurrence risk than from the anterior circulation (30), and the intracranial aneurysm (IA) could increase the hemorrhagic risk for MMD patients (31). Although they were not identified as risk factors of future strokes, our study found that hemorrhagic-type MMD patients with IA showed relatively higher recurrent stroke risks than those without IA (15.2 vs. 12.0%). Additionally, the ischemic type of TIA and cerebral infarction directly reflects the severity of stenosis and/or occlusion in the intracranial arteries, while patients with an initial symptom of lacunar infarction show a very low recurrent stroke risk, which is similar to the report from Zhao et al. (32), suggesting that lacunar infarction may act as a biomarker associated with a favorable long-term prognosis of low-risk recurrence. Moreover, our study has also found that surgical revascularization was an effective strategy for adult MMD in preventing future strokes (conservative *vs*. antiplatelet *vs*. surgical = 13.5 *vs*. 8.1 *vs*. 4.1%; Log-rank test: Chi-square = 5.404, *p* = 0.02; Supplementary Figures 1A,B). Even though the use of antiplatelet agents in the treatment of MMD remains controversial, it was interesting that antiplatelet therapy reduced the future stroke risk compared with surgical and conservative treatments for ischemic-type patients in our study (conservative *vs*. antiplatelet *vs*. surgical = 3.8 *vs*. 8.5 *vs*. 14.5%; Log-rank test: Chi-square = 4.717, *p* = 0.03; Supplementary Figures 1C,D). Recent evidence has demonstrated that luminal thrombosis is a pathological feature of MMD and contributes to steno-occlusive changes in the distal ICA (1). In addition, antiplatelet agents have been widely applied for preventive management in acute ischemic stroke and TIA (33, 34). Thus, more clinical studies should be performed to investigate the efficacy and safety of antiplatelet agents in the future.

In contrast to previous studies, our study observed that a family history of MMD and diabetes mellitus were significantly associated with future strokes. The proportion of patients with a family history of MMD was ~ 5.6%, which is consistent with the report from Duan et al. (5), indicating that a genetic factor might play a role in the pathogenesis of MMD. Although no obvious differences were apparent between MMD patients and the general population, diabetes mellitus was significantly correlated with severe cerebrovascular disease and contributed to cerebral neovascularization. Ren et al. (35) report that adult MMD patients with diabetes mellitus acquire better cerebral perfusion after revascularization. However, one different result from an observational study with a large sample size demonstrates that diabetes mellitus acts as a risk factor for recurrent strokes in young adults with MMD after revascularization (36). Therefore, the effect of diabetes mellitus in patients with MMD remains unclear. Our study supports diabetes mellitus as a risk factor for recurrent stroke, regardless of the treatment choice. Although an invalidated hypothesis suggests that diabetes mellitus is associated with elevated growth factors and cytokines causing the formation of collateral circulation to improve the blood supply, the major problem for patients with MMD is the chronic steno-occlusive change of ICAs causing the cerebral hypoperfusion and that abnormal angiogenesis could deteriorate the cerebral ischemia. Thus, additional randomized case-control studies are necessary to evaluate the predictive effectiveness of these factors in the future.

Several clinical benefits are associated with the application of the predictive nomogram. The nomogram can be easily used to calculate the individual future stroke risk and provide important treatment recommendations in clinical practice. For patients with a low risk of future stroke, blood sugar monitoring and a long-term medical follow-up should be recommended. For high-risk patients, personalized therapy is required to provide more appropriate treatment to reduce the risk level and improve the prognostic outcome. In addition to an outpatient follow-up and blood sugar control, therapeutic changes are necessary for patients. With regard to hemorrhagic-type MMD, surgical revascularization is employed to prevent recurrent stroke and improve neurological dysfunctions (10–13). For ischemic-type MMD, antiplatelet therapy is inferior to surgical revascularization while superior to conservative management for changing the blood supply and preventing future stroke events (37). Revascularization surgery is capable of reducing the recurrence of future stroke for patient with hemorrhagic MMD, but it has not shown an advantage over conservative treatment for patients with ischemic MMD (14, 38). Many patients are not willing to undergo surgery because of their mild symptoms and/or high operative risks in clinical practice. Although antiplatelet therapy remains controversial, our study shows a positive result that proves its capacity, especially for patients with the initial symptom of cerebral infarction. Additionally, this nomogram provides prognostic information for doctors to identify high-risk patients receiving antiplatelet agents and prompts the selection of revascularization as a replacement treatment. With regard to asymptomatic MMD, revascularization and antiplatelet therapy are also controversial, and clinical studies demonstrating the effectiveness of such treatments are not abundant. Thus, doctors can encourage these treatments as supplementary therapy to prevent future strokes.

The strengths of the current study include a relatively larger number of enrolled patients, the long-term follow-up, and the broad scope of the clinical data collection from seven high-level hospitals across China. However, several limitations exist in this study. The genomic characteristics were not included in our analysis. Some genome-wide and locus-specific association studies identified that RNF213 polymorphism was an important genetic risk factor for MMD in East Asian population (39) which may influence the clinical phenotypes of MMD (40). Several meta-analysis studies shown that RNF213 p.4810K significantly and markedly increased MMD risk (Odds ratio = 60–100) (41–43). Even though the precise mechanism of the RNF213 variant in MMD pathogenesis is not clear (44), genetic epidemiological studies have rigorously identified RNF213 p.R4810K as a risk factor in MMD patients. In addition, this variant was reported to increase the risk of ischemic stroke due to large-artery atherosclerosis (45). Certain previous studies have reported that ~1% of the general population were unaffected genetic carriers in the East Asian population (4), but the gene was also associated with other diseases (46, 47), suggesting that RNF213 might not be a specific gene for MMD. However, the incidence rate of RNF213 polymorphism in MMD was proven relatively higher (0.41 per 100,000, 95% CI: 0.28–0.54) in the Chinese population and the effect of this variant was very important for MMD patients (4). Thus, an insufficient survey of RNF213 polymorphism is a limitation in this study and RNF213 variant should be investigated in the future. Furthermore, some potential risk factors, such as vascular endothelial growth factor (VEGF) and matrix metalloprotein (MMP) status, which are not routinely evaluated, were not included. In contrast to a previous study, computed tomography perfusion (CTP) imaging and positron emission tomography (PET) scans for hemodynamic reserve and cerebral perfusion are not conducted in most Chinese patients due to their high cost and medical insurance limitations. Moreover, the proportion of patients who underwent surgical revascularization was lower than previous studies; in addition, the rate was substantially lower in patients from western China than in those from southern coastal China. In addition to the presence of mild symptoms and fears regarding surgery, education level and economic income might also result in this difference. Based on this study, the individual risk score could be calculated using this prediction model as follows: risk score = diabetes mellitus × 1.12566516 + a family history of MMD × 2.04512987 + a past history of TIA or stroke × 1.50137252 + clinical manifestation × 0.5129745 + treatment × (−0.6259136). Since the individual risk score was a skewed distribution, the median value (M) and interquartile range (IQR) were used to describe the normal range of the risk score in this study (M = 1.68, IQR = 0.36–1.85). Importantly, since our prediction model was entirely based on Chinese patients, the generalizability of this risk score in the areas outside of China is limited. However, these clinical features could be easily recorded in clinical practice, so our study may provide scientists outside of China with a new and viable approach in further studies. It is possible that the prediction models of other ethnic groups differ from those of the Chinese population. Ultimately, some aspects are not completely known, such as environmental factors and other conditions.

In conclusion, our study presents a novel nomogram incorporating several major risk factors with relatively good accuracy. The nomogram can be conveniently used to help neurologists and neurosurgeons evaluate the individual risk of a future stroke event and provide useful treatment recommendations for adult MMD patients in clinical practice.

## Data Availability Statement

The original contributions presented in the study are included in the article/Supplementary Material, further inquiries can be directed to the corresponding author/s.

## Ethics Statement

The studies involving human participants were reviewed and approved by the independent ethics committee of the First Affiliated Hospital of Sun Yat-sen University and each participating medical center's ethics committee (number: [2020]138). Written informed consent for participation was not required for this study in accordance with the national legislation and the institutional requirements.

## Author Contributions

FY, TW, and HY designed the study, drafted the manuscript, and contributed to the discussion. JL analyzed the data. HL, TG, XZ, TY, LJ, and XW collected the data. WS and QL designed the study, reviewed the manuscript, and contributed to the discussion. All authors contributed to the article and approved the submitted version.

## Conflict of Interest

The authors declare that the research was conducted in the absence of any commercial or financial relationships that could be construed as a potential conflict of interest.

## Supplementary Material

Supplementary Figure 1 The survival analysis of future strokes in adult patients with moyamoya disease (MMD). **(A)** The cumulative stroke-free survival in all the MMD patients (Log-rank test: Chi-square = 5.40, *p* = 0.02). **(B)** The cumulative risk in all the MMD patients. **(C)** The cumulative stroke-free survival in the ischemic-type MMD patients (Log-rank test: Chi-square = 4.72, *p* = 0.03). **(D)** The cumulative risk in the ischemic-type MMD patients.

Supplementary Table 1 The original data of 450 adult patients with moyamoya disease.

Supplementary Table 2 The training set of 300 adult patients with moyamoya disease.

Supplementary Table 3 The test set of 150 adult patients with moyamoya disease.

Supplementary Table 4 The original data of 187 adult patients with ischemic-type moyamoya disease.

Supplementary Table 5 The original data of 216 adult patients with hemorrhagic-type moyamoya disease.
