# Supplementary figures and images for: Development and Validation of a Nomogram to Predict the Individual Future Stroke Risk for Adult Patients With Moyamoya Disease: A Multicenter Retrospective Cohort Study in China
Source: Front Neurol. 2021 May 13;12:669025. doi: 10.3389/fneur.2021.669025 (PMC8155507; doi:10.3389/fneur.2021.669025)

**A**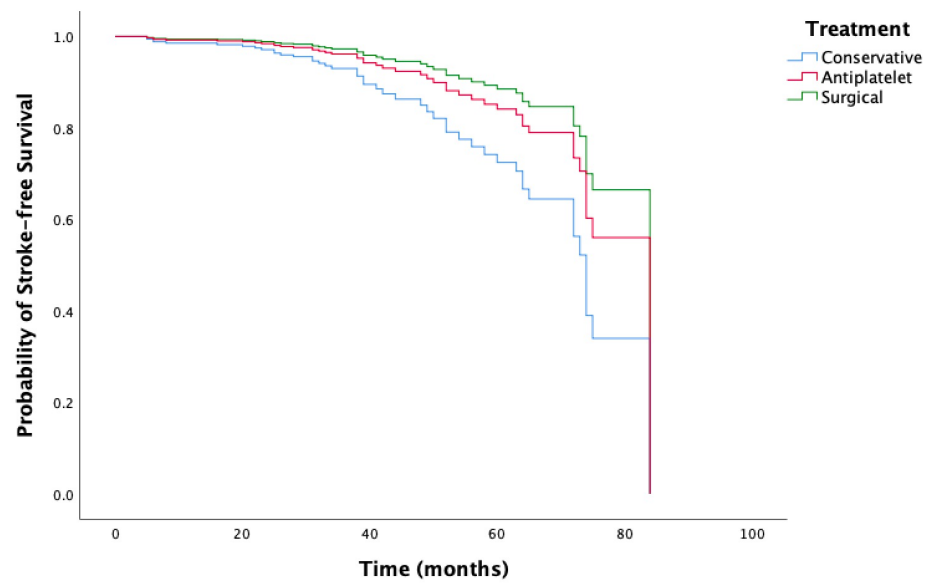**B**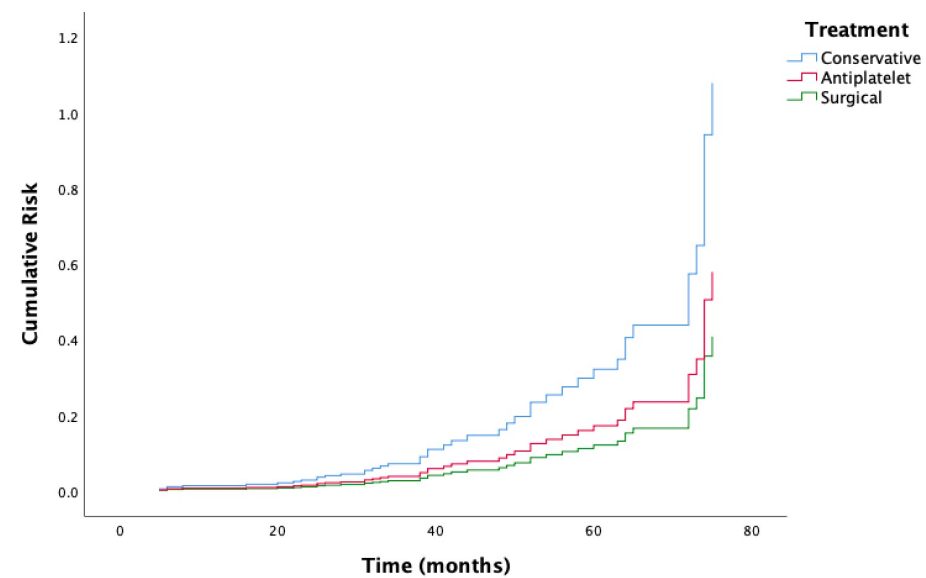**C**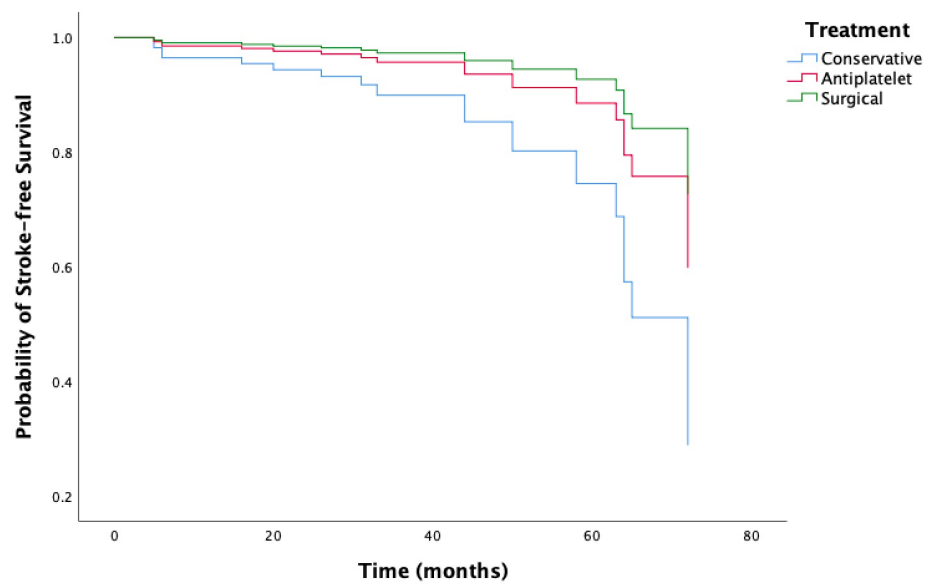**D**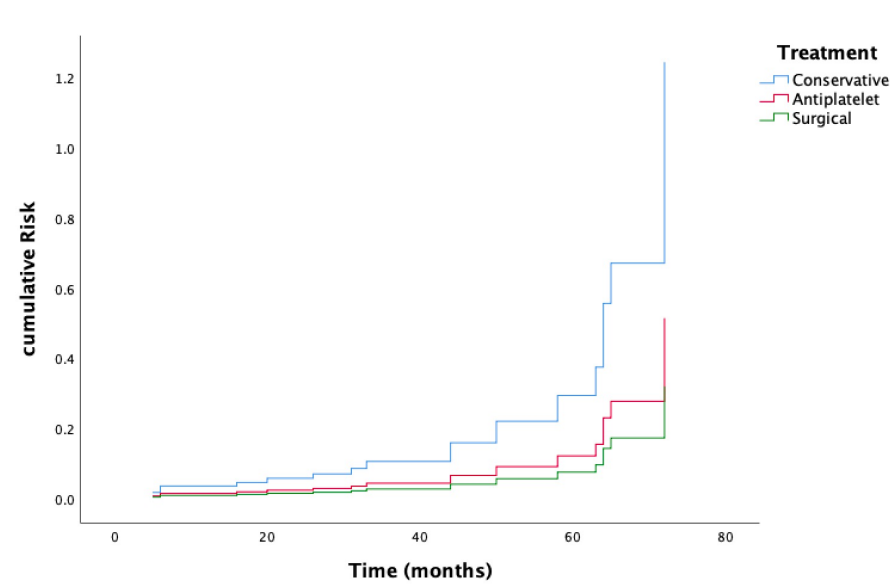

Supplement: Supplementary Figure 1 — The survival analysis of future strokes in adult patients with moyamoya disease (MMD). (A) The cumulative stroke-free survival in all the MMD patients (Log-rank test: Chi-square = 5.40, p = 0.02). (B) The cumulative risk in all the MMD patients. (C) The cumulative stroke-free survival in the ischemic-type MMD patients (Log-rank test: Chi-square = 4.72, p = 0.03). (D) The cumulative risk in the ischemic-type MMD patients. [file Image_1.PDF]
